# Possible Iodine-Induced Thyrotoxicosis in a Previously Healthy Adolescent following Administration of Iodinated Contrast Media

**DOI:** 10.1155/2021/5930515

**Published:** 2021-11-23

**Authors:** Jennifer Apsan, Zoltan Antal

**Affiliations:** Department of Pediatrics, Division of Endocrinology Weill Cornell Medicine, New York Presbyterian Hospital, New York, NY, USA

## Abstract

*Introduction*. Jod-Basedow Syndrome refers to a paradoxical phenomenon in which large loads of iodine can cause hyperthyroidism. It is most commonly seen in populations already at risk for thyroid disease or those with underlying kidney disease. *Case Presentation*. We present a case of an acutely ill 17-year-old boy with symptomatic hyperthyroidism following an iodinated contrast CT scan to rule out appendicitis. *Discussion/Conclusion*. This case underscores the importance of recognizing this phenomenon even in the pediatric population and in those with no preexisting history of thyroid disease. Course complications including bronchospasm, hypertension, transaminitis, and bilateral palmar desquamating rash are rare and highlight the complexities involved in the disease state and in managing side effect profiles of treatment.

## 1. Introduction

Iodine is an essential mineral, necessary for optimal thyroid function. Iodine is commonly found in a variety of foods, such as iodized salt and seafood, and in routinely utilized household items, including nutritional supplements and skin cleaners. In medical settings, iodine is a component of various medications (amiodarone and expectorants), iodine-based swabs used for skin cleaning prior to invasive procedures, and iodinated contrast media (ICM). ICM is an increasingly common source of potential iodine toxicity as CT scans have become an increasingly popular imaging modality in the United States [1]. Recommended adult daily iodine intake is typically set at about 150 *μ*g per day by the Institute of Medicine, United Nations Children's Fund (UNICEF), World Health Organization, and the International Council for the Control of Iodine Deficiency Disorders (ICCIDD) [2]. Contrast media is known to contain about 13,500 *μ*g of free iodine and 15–60 g of bound iodine [3, 4]. Typically, the physiological response to these large iodine loads is a transient hypothyroid state, induced by inhibiting the thyroid peroxidase enzyme involved in thyroglobulin iodination in a phenomenon known as the Wolff–Chaikoff effect. Eventually, equilibrium ensues as downregulation of the Na-I transporters which permit less iodine into the thyroid gland, thus allowing the gland to return to normal function by escaping the Wolff–Chaikoff effect. The escape or adaptation phenomenon can occur in as quickly as 48 hours and allows for adaptation to a high iodide environment without risk of thyroid dysfunction [5, 6]. Inability to escape from this inhibitory effect of large iodine doses can result in clinical or subclinical hypothyroidism. This has been described largely in fetuses, newborns, patients with underlying chronic disease, patients with autoimmune thyroiditis, or in those previously treated with radioimmunoassay (RAI), surgery, or antithyroid drugs, but has also been described in some with no known underlying conditions [7, 8]. In some people, a paradoxical hyperthyroid state is induced after administration of a large iodine load. Typically, this is seen in those with autoimmune thyroiditis, prior thyroid surgical history, prior goiter formation, Graves' disease, or kidney disease, which can impact iodine excretion.

Mainstays of treatment for hyperthyroidism include withdrawal of the offending agent, antithyroid drugs, beta blockade to decrease sympathetic tone, and steroids [5]. Multiple cases of iodine-induced hyperthyroidism are described in the adult population. Many of these patients have underlying thyroid or kidney disease which can predispose them to a state of iodine-induced hyperthyroidism [9–11]. This phenomenon is not described well in the pediatric population. Here, we describe the presentation, diagnosis, and management of a 17-year-old boy with presumed iodine-induced hyperthyroidism.

## 2. Case Presentation

A previously healthy 17-year-old adolescent presented to the emergency department with 3-4 days of acute and chronic right lower quadrant abdominal pain. He was sent for a protocoled contrast CT scan (78 ml Omnipaque 300) that was suggestive of acute appendicitis. He was taken to the operating room, on postcontrast day 1, where his appendix was removed. Intraoperatively, he experienced a brief episode of bronchospasm with complete resolution and successful extubation following epinephrine administration. The patient was found to be positive for rhinovirus/enterovirus at that time. On postoperative day 2, the patient had an acute episode of flushing, fatigue, fevers, and tachycardia. Infectious workup was pursued, and antibiotics were started. On postoperative day 4, flushing and tachycardia (HR 100–110 bpm) persisted, and further laboratory evaluation was notable for an elevated serum free T4 level of 4.1 ng/dL (reference values: 0.9–1.8 ng/dL) and a serum TSH level of <0.0008 uiu/ml (reference values: 0.6–4.8 uiu/ml), confirming a diagnosis of hyperthyroidism. Burch–Wartofsky score at the time of symptoms was 15, indicating a low risk for thyroid storm. Physical exam revealed that the patient was extremely fatigued, weak, and tremulous. There was no exophthalmos or lid lag, and no palpable thyromegaly or thyroid nodules. Further evaluations revealed negative thyroid stimulating immunoglobulin (TSI), antithyroglobulin, and antithyroperoxidase antibody levels. A significantly elevated serum thyroglobulin level was noted, 274 ng/ml (reference values: 0.8–29.4 ng/ml). Thyroid ultrasound was normal with no evidence of thyroid enlargement, hyperemia, or heterogeneity of the gland. Thyroid scintigraphy was considered but deferred secondary to the recent iodine load given for CT scan.

**Figure 1:**
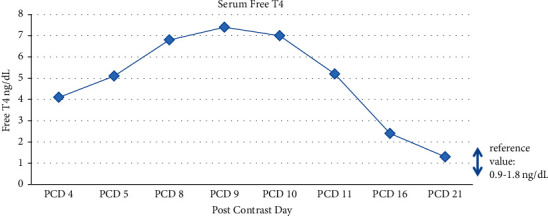
Serum Free T4 level in case study patient.

Methimazole therapy was initiated but appeared to be inadequate as a monotherapy despite increasing dosing, as there was a rapid worsening of clinical symptoms and biochemical hyperthyroidism (Table 1/Figure 1). A mild transient transaminitis was also noted, raising concerns over increasing the methimazole dose. Steroids and propranolol were added to the regimen on postcontrast day 9 which resulted in a rapid and steady decline of serum thyroxine levels over the next 7 days. One week after discharge and 12 days after the initiation of therapy, methimazole was weaned and discontinued secondary to a significant worsening transaminitis and bilateral palmar desquamating rash. Steroid therapy was also stopped after 8 days of treatment due to episodic hypertension. Further evaluation of the potential cause of transaminitis revealed an acute EBV infection. The transaminitis significantly improved within the subsequent 10 days, and thyroid function continued to remain within normal limits. Three months later, transaminitis fully resolved.

Serum iodine studies were obtained during the hospitalization. After contrast day 10, serum iodine levels were found to be 181 ug/L (reference values: 90–92 ug/L), which fully normalized to 79 ug/L (reference values: 52–109 ug/L) on postcontrast day 22, at which time thyroid function tests normalized as well.

## 3. Discussion/Conclusion

**Figure 2:**
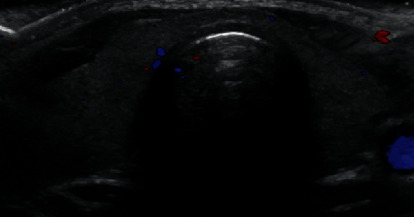
Thyroid ultrasound on post contrast day 7 reveals homogeneous and non enlarged thyroid gland measuring Isthmus: 0.2 cm, Right lobe: 2.3 × 4.3 × 0.9 cm Left lobe: 1.3 × 4.3 × 1.1 cm. Echogenicity is within normal limits with no increased vascularity.

Our patient's clinical and laboratory data confirmed a diagnosis of acute, transient hyperthyroidism. The differential for transient thyroiditis in an adolescent includes Hashitoxicosis (hyperthyroidism caused by inflammation associated with Hashimoto's thyroiditis) and subacute thyroiditis in the setting of acute inflammation secondary to trauma or viral infection. Although autoimmune thyroiditis is the most common underlying cause of acute hyperthyroidism, our patient's autoantibody titers were negative, and thyroid ultrasound findings were not consistent with a diagnosis of autoimmune thyroiditis. He also had no evidence of inflammation, enlargement, or increased vascularity on thyroid ultrasound (Figure 2), and clinically, he had no evidence of pain or enlargement to his thyroid gland, making traumatic injury to thyroid from his intubation or virally induced hyperthyroidism unlikely. A less common cause of acute transient hyperthyroidism is excess iodine administration in a phenomenon known as Jod-Basedow syndrome [5]. The time course of our patient's presentation shortly following administration of iodinated contrast supports iodine-induced hyperthyroidisim or Jod-Basedow syndrome as the most likely cause. At a dose of 78 ml of Omnipapague 300, our patient was exposed to approximately 23 grams organic iodine; many folds more than the International Council for the Control of Iodine Deficiency Disorders (ICCIDD) recommended a tolerable upper level of 1,100 *μ*g per day in adults [1, 12] Significantly increased thyroglobulin levels well above the normal range, as were seen in our patient, support overactivity of the thyroid gland. Thyroglobulin levels are thought to correlate with iodine exposure and may be a potential biomarker of iodine overload [13, 14].

Although the diagnosis of Jod-Basedow Syndrome is not typically dependent on confirmed elevation in serum iodine levels, our patient's elevated iodine levels correlated directly with the change in thyroid functions, further supporting the diagnosis [1]. Although other case reports have noted even higher iodine levels, our initial serum measurement was obtained 10 days after contrast administration and 7 days following initial diagnosis of hyperthyroidism. Thus, the iodine levels were presumed to be higher at the onset of disease.

Jod-Basedow syndrome typically occurs in those who have underlying thyroid disease such as autoimmune thyroiditis, goiter formation, or in those with underlying kidney disease as iodine is renally excreted. Our patient had previously documented routinely obtained thyroid function tests for an unrelated reason 2 years prior to this hospitalization (Table 1), decreasing our suspicion of prior underlying thyroid disease. Furthermore, the documented negative thyroid antibody status and normal renal function during the admission further excludes the typical underlying risk factors. We suspect that his underlying rhinovirus/enterovirus and EBV infections may have precipitated this thyroid response to iodinated contrast. The connection between viral infection and impaired thyroid function is well documented [15]. Specifically, EBV has been shown to have immunomodulatory effects and has been linked to autoimmune thyroid disease [16, 17]. In our patient, the exact mechanism of interaction between vial infection and response to iodine is not fully understood.

The reported timeline of contrast-induced hyperthyroidism in other case reports is variable, typically occurring from days to weeks following contrast administration [3, 14, 18]. However, there are reports of hyperthyroidism occurring as early as postcontrast day 1 [4]. While most cases of Jod-Basedow syndrome present as subclinical hyperthyroidism without clinical symptoms, our patient was overtly symptomatic with tremulousness and tachycardia. It not fully clear why the range of reactions to iodine exposure is so varied. Recent data point to a possible genetic predisposition [19]. Bronchospasm is not clearly associated with Jod-Baslow syndrome, and it remains unclear to date if this event in our patient was related to his thyroid disease or a reaction to his anesthesia [3, 4, 18].

This case also highlights the importance of managing side effects once medication is deemed necessary. Our patient developed both transmaminitis and hypertension. Transaminitis poses a clinical conundrum, as it has been described both as part of the natural history of hyperthyroidism [20] as well as a potential adverse effect of thionamides. In our case, the differential diagnosis additionally included EBV infection. Our decision to closely observe and continue methimazole therapy led to a successful outcome, but required a multidisciplinary effort involving other medical disciplines as well as the patient and patient's family. Steroid treatment was initiated in an effort to decrease peripheral conversion of T4 to T3 and to potentially avoid requiring increased Methimazole dosing. Thyroid function tests improved significantly once steroids were started, but our patient became significantly hypertensive at outpatient follow-up, and steroids were discontinued 6 days later. Given the uncertainty of the timeline and duration of our patient's hyperthyroidism, coupled with his significant clinical symptoms, we believe the steroid therapy was a valuable addition to the treatment regimen and was associated with significant clinical and biochemical improvement.

This case highlights the complexity of thyroid physiology and the importance of considering Jod-Basedow syndrome as a differential diagnosis even in previously healthy populations with no overt overlying risk factors. It also highlights the complexities involved in managing side effect profiles of treatment and the need for close follow-up. Although the majority of reported cases are in adults, it is important that pediatric providers are aware of the risk of this condition occurring in children as well.

## Figures and Tables

**Table 1:** Thyroid function tests.

	12/28/17+	2/23/20 PCD 4	2/24/20 PCD 5^*∗*^	2/27/20 PCD 8	2/28/20 PCD 9^*∗∗*^	2/29/20 PCD 10	3/1/20 PCD11	3/6/20 PCD16^*∗∗∗*^	3/11/20 PCD 21^*∗∗∗∗*^
TSH (uIU/ml) 0.6–4.8	1.165	<0.0008	<0.0008	0.0009	0.008	0.009	0.008	0.011	0.822
Free T4 (ng/dL) 0.9–1.8 (ng/dL)	1.0	4.1	5.1	6.8	7.4	7.0	5.2	2.4	1.3
Total T4 (ug/dL) 4.5–10.9		15.0	16.5	26.9	26.3	24.7	21.5	8.1	6.4
Total T3 (ng/mL) 0.6–1.8		1.79	1.81	4.06	3.90	2.76	1.91	0.76	1.00
TSI (IU/L) <0.54IU/L			<0.10						
TPO ab (IU/ml) 0.0–9.0			<0.3						
Tg ag (IU/ml) 0.0–4.0			<0.9						

PCD: Post contrast day, +available as baseline from prior GI appointment for poor weight gain, ^*∗*^methimazole started, *∗∗*hydrocortisone 100 mg q 8/propranolol 40 mg q 8 hours started, ^*∗∗∗*^methimazole stopped/Hydrocortisone stopped, ^*∗∗∗∗*^propranolol stopped.

## Data Availability

The data used to support the findings of this study from extensive medical literature search are included within the article and cited in the references.
